# ENCODE Tiling Array Analysis Identifies Differentially Expressed Annotated and Novel 5′ Capped RNAs in Hepatitis C Infected Liver

**DOI:** 10.1371/journal.pone.0014697

**Published:** 2011-02-16

**Authors:** Milan E. Folkers, Don A. Delker, Christopher I. Maxwell, Cassie A. Nelson, Jason J. Schwartz, David A. Nix, Curt H. Hagedorn

**Affiliations:** 1 Department of Medicine, University of Utah, Salt Lake City, Utah, United States of America; 2 Huntsman Cancer Institute, University of Utah, Salt Lake City, Utah, United States of America; 3 Department of Surgery, University of Utah, Salt Lake City, Utah, United States of America; 4 Department of Experimental Pathology, University of Utah, Salt Lake City, Utah, United States of America; Washington University, United States of America

## Abstract

Microarray studies of chronic hepatitis C infection have provided valuable information regarding the host response to viral infection. However, recent studies of the human transcriptome indicate pervasive transcription in previously unannotated regions of the genome and that many RNA transcripts have short or lack 3′ poly(A) ends. We hypothesized that using ENCODE tiling arrays (1% of the genome) in combination with affinity purifying Pol II RNAs by their unique 5′ m^7^GpppN cap would identify previously undescribed annotated and unannotated genes that are differentially expressed in liver during hepatitis C virus (HCV) infection. Both 5′-capped and poly(A)+ populations of RNA were analyzed using ENCODE tiling arrays. Sixty-four annotated genes were significantly increased in HCV cirrhotic as compared to control liver; twenty-seven (42%) of these genes were identified only by analyzing 5′ capped RNA. Thirty-one annotated genes were significantly decreased; sixteen (50%) of these were identified only by analyzing 5′ capped RNA. Bioinformatic analysis showed that capped RNA produced more consistent results, provided a more extensive expression profile of intronic regions and identified upregulated Pol II transcriptionally active regions in unannotated areas of the genome in HCV cirrhotic liver. Two of these regions were verified by PCR and RACE analysis. qPCR analysis of liver biopsy specimens demonstrated that these unannotated transcripts, as well as IRF1, TRIM22 and MET, were also upregulated in hepatitis C with mild inflammation and no fibrosis. The analysis of 5′ capped RNA in combination with ENCODE tiling arrays provides additional gene expression information and identifies novel upregulated Pol II transcripts not previously described in HCV infected liver. This approach, particularly when combined with new RNA sequencing technologies, should also be useful in further defining Pol II transcripts differentially regulated in specific disease states and in studying RNAs regulated by changes in pre-mRNA splicing or 3′ polyadenylation status.

## Introduction

Microarray based gene analyses have provided a new approach to identifying disease-specific changes in gene expression. This approach has improved the understanding of molecular pathobiology by identifying genes that are differentially regulated in selected diseases and by identifying biomarkers for disease states or responses to therapy. Successful examples of this approach include identifying subtypes of diffuse large B-cell lymphomas with different prognoses and increased expression of the zeta-chain (TCR) associated 70 kDa protein kinase (*ZAP70*) in chronic lymphocytic leukemia (CLL) which is a predictor of the clinical course [1], [2], [3]. Examples in stratifying breast cancer include identifying a panel of transcriptional changes predicting distant metastasis in lymph-node-negative patients, predicting the response to specific therapies and survival of patients [4], [5], [6], [7], [8], [9]. Progress towards stratifying patients with colon cancer using gene expression signatures was recently reported [10].

In hepatitis C infection, gene array analyses in chimpanzees identified host responsive gene pathways in acute and chronic hepatitis C infection [11]. Gene array studies have also been used to identify potential biomarkers of hepatitis C infection, interferon regulated genes which predict the response to therapy, and gene expression patterns which predict early progression to fibrosis in liver transplant recipients [12], [13], [14]. Unique gene expression profiles were also identified which distinguish alcoholic liver disease and hepatitis C as well as hepatitis B and hepatitis C infection [15], [16]. More recent data suggests that the hepatitis C virus may regulate host non-coding RNAs (i.e., miRNAs) to promote viral replication [17].

One limit of standard oligonucleotide or cDNA microarrays is that they require prior knowledge of the sequence of the RNA that will be measured. In addition, many arrays are purposefully biased to interrogate sequences originating from 3′ ends of mRNA encoded by the 3′ terminal exon of previously annotated genes. Although these methods measure RNA transcripts from well-annotated protein coding genes and selected ncRNAs, they do not generally provide information regarding pre-mRNAs and most non-coding RNAs that can be important in physiological or pathological processes. Genomic tiling arrays or next generation sequencing provide a useful alternative for quantifying and characterizing transcription across the genome without requiring prior knowledge of gene or RNA transcript sequences. Tiling arrays are designed to cover the entire genome, selected chromosomes or contigs of the genome. Recent gene expression experiments using ENCODE (**Enc**yclopedia **o**f **D**NA **E**lements) tiling arrays, representing 1% of the human genome, have demonstrated that much of the genome is transcribed and that most nucleotides appear to be present in at least some form of RNA transcript [18]. A recent study in *Schizosaccharomyces pombe* provides further evidence for extensive sense and antisense transcription [19]. The nature of these RNAs, the RNA polymerase responsible for their transcription and their biological function remain unknown.

Previous ENCODE studies analyzed polyadenylated [poly(A)+] RNA, but recent data suggests that many RNAs in yeast and mammals either lack or have short 3′ poly(A) ends and are underrepresented or absent in microarray analyses using poly(A)+ RNA [18], [20]. To enhance the selection of Pol II transcripts we have developed an efficient method of purifying RNA polymerase II (Pol II) transcripts regardless of their 3′ polyadenylation status. Pol II RNAs have a 5′ m7GpppN cap added enzymatically to their 5′ ends during the pausing phase of transcription [21], [22]. Our approach purifies Pol II transcripts by binding their 5′ caps with a high-affinity variant of the RNA cap binding protein (eIF4E_K119A_) [20], [23]. When compared to standard poly(A)+ dependent purifications the yield of 5′ capped RNA is 2–3 fold greater from the same quantity of total RNA starting material, suggesting that poly(A)+ purification does not recover all capped Pol II RNAs [20].

To date, no gene expression studies on hepatitis C infected liver have been performed using tiling array analyses such as ENCODE. The goal of this study was to identify differentially expressed annotated genes and novel RNAs in hepatitis C infected liver that would not typically be recorded by analyzing poly(A)+ RNA with standard gene expression arrays. In this study, we utilized 5′ capped and poly(A)+ RNA populations isolated from control and chronically infected hepatitis C (HCV) cirrhotic human liver biospecimens using ENCODE tiling arrays to measure differential expression of Pol II RNAs. Differentially expressed RNAs identified in this analysis were then measured by real-time PCR (qPCR) in additional control, mild chronic hepatitis C (no fibrosis) and chronically infected hepatitis C cirrhotic biospecimens.

## Results

**Figure 1:**
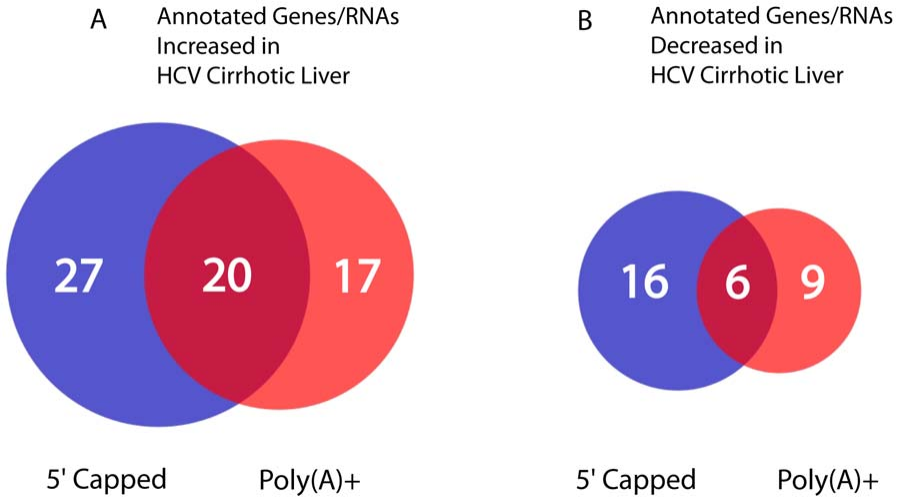
Differentially expressed genes in HCV cirrhotic versus control liver when analyzing 5′ or 3′ isolated RNA. **Panel** **A.** The number of genes with RNA levels that were increased in hepatitis C (HCV) cirrhotic as compared to control liver in the 5′ capped, poly(A)+, and both RNA populations are shown. **Panel** **B.** The number of genes with RNA levels that were decreased in HCV cirrhotic as compared to control liver in 5′ capped, poly(A)+, or both RNA populations are shown. RNA transcripts were isolated from HCV cirrhotic and control liver with the 5′ cap and poly(A)+ dependent purifications described in Methods. Four experimental replicates of HCV cirrhotic and control liver were used for this analysis. cDNA was synthesized using random hexamers and used to prepare probes (Methods). RNA transcript expression was measured by averaging fluorescent signal intensity on Agilent ENCODE gene arrays for each sample. Annotated genes with ≥1.5 fold changes and Bonferroni corrected p values <0.05 in HCV cirrhotic as compared to control liver were included in this figure.

The ENCODE tiling array analysis of 5′ capped RNA identified 47 annotated genes with increased expression (fold change >1.5, Bonferroni adjusted p value <0.05) (Figure 1A) and 22 genes with decreased expression in a chronic hepatitis C (HCV) cirrhotic as compared to an uninfected control liver specimen (Figure 1B). Analysis of poly(A)+ RNA identified 37 genes with increased expression and 15 genes with decreased expression in HCV cirrhotic as compared to control liver. Twenty of the upregulated genes and six of the downregulated genes were identified in both 5′ capped and poly(A)+ RNA populations (Figure 1). Of note, 8 out of 17 upregulated genes (47%) and 2 out of 9 down-regulated genes (22%) unique to poly(A)+ RNA did have a statistically significant expression difference (p<0.05) in 5′ capped RNA, but were excluded from our list because their fold change did not meet the inclusion criteria (<1.5). None of 27 upregulated or 17 downregulated genes unique to 5′ capped RNA were found to have significant differential expression in the poly(A)+ RNA (see Tables S1, S2, S3, S4, S5, S6). This is likely due to less variation in signal intensity observed with 5′ capped RNA among experimental replicates as compared to poly(A)+ RNA (the average SEM per gene was 28.1 for 5′ capped; poly(A)+ average SEM per gene was 77.2).

**Figure 2:**
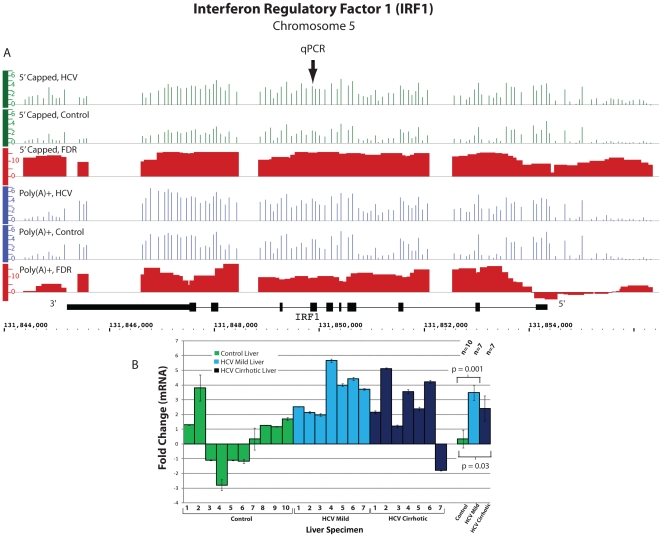
Differential expression of *IRF1* in HCV cirrhotic as compared to control liver. **Panel A.** Expression of *IRF1* as measured by signal intensity on ENCODE tiling arrays is displayed using the Integrated Genome Browser (IGB). The y-axis represents the log2 transformation of the normalized signal divided by the background signal on arrays and each bar represents the normalized signal intensity of probes hybridized to 60mer targets tiled across the gene region (Methods). Genomic regions that are tiled yet lack a signal are indicated by a baseline tick mark. Absence of a tick mark indicates no probes in that region. Gene structure, orientation, and chromosomal location are shown in black. Average RNA expression based on the analysis of 5′ capped RNA isolated from HCV cirrhotic and control liver is depicted in green for both HCV cirrhotic and control liver. Average RNA expression using poly(A)+ RNA is depicted in blue. Differences in expression between HCV cirrhotic and control liver for poly(A)+ and 5′ capped RNA populations are depicted as window level false discovery rates in red (-10Log_10_ FDR) (see Methods). A transformed FDR of ≥13 (represents an untransformed FDR ≤0.05 or 5 false positives out of 100) was considered statistically significant. **Panel** **B.** qPCR was performed as described in Methods. Triplicate samples from seven HCV cirrhotic, seven mild HCV (no fibrosis), and ten control livers were analyzed. HCV Cirrhotic 1 and Control 1 refer to the original samples used for the ENCODE tiling array analysis. The mean ± SEM fold change for each specimen analyzed is shown and the location of the qPCR primers is indicated by qPCR in Panel A. P-values were calculated using the Student's t-test.

Thirteen of the 64 genes (20%) found to be upregulated in HCV cirrhotic liver were identified by GO-term enrichment analysis to have biologic functions related to the immune response (Table 1) [24], [25]. Several of these genes were selected for qPCR analysis of liver tissue from multiple patients, including interferon regulatory factor 1 (*IRF1*), a transcription factor involved in the interferon response to HCV infection *in-vitro* [26]. On ENCODE analysis, *IRF1* was found to be upregulated in HCV cirrhotic liver in 5′ capped RNA only (fold change 2.1, p = 0.03, Figure 2A). qPCR analysis of *IRF1* in multiple patient samples confirmed the ENCODE findings of increased expression in HCV cirrhotic liver (n = 7) compared to control liver (n = 10) (fold change 2.4, p = 0.03, Figure 2B). Percutaneous liver biopsies (n = 7) from patients with chronic hepatitis C with mild inflammation and no fibrosis (Metavir grade 1, stage 0) were also analyzed and showed a significant increase in *IRF1* expression (fold change 3.5, p = 0.001, Figure 2B). These patients were not being treated with recombinant interferon at the time of liver biospecimen acquisition.

**Table 1:** Differentially Expressed Genes Associated with Immune Response.

Gene Name (Ensembl)	Description	Isolation Technique	Mean Signal Intensity HCV Liver	Mean Signal Intensity Control Liver	Fold Change	Bonferroni p Value
**Upregulated only in 5′ capped RNA**						
*IRF1	Interferon regulatory factor 1 (IRF-1)	**5′ Capped RNA**	**674**	**323**	**2.09**	**0.033**
		Poly(A)+ RNA	2087	1363	1.53	51.505
LILRA1	Leukocyte immunoglobulin-like receptor subfamily A member 1 Precursor	**5′ Capped RNA**	**192**	**121**	**1.58**	**<0.001**
		Poly(A)+ RNA	219	171	1.28	18.386
LILRA4	Leukocyte immunoglobulin-like receptor subfamily A member 4 Precursor	**5′ Capped RNA**	**106**	**64**	**1.64**	**<0.001**
		Poly(A)+ RNA	126	102	1.24	7.956
*LILRB2	Leukocyte immunoglobulin-like receptor subfamily B member 2 Precursor	**5′ Capped RNA**	**756**	**359**	**2.1**	**0.015**
		Poly(A)+ RNA	1186	756	1.57	7.591
*LILRB4	Leukocyte immunoglobulin-like receptor subfamily B member 4 Precursor	**5′ Capped RNA**	**191**	**92**	**2.06**	**<0.001**
		Poly(A)+ RNA	378	246	1.54	0.388
RFX5	DNA-binding protein RFX5	**5′ Capped RNA**	**292**	**174**	**1.68**	**<0.001**
		Poly(A)+ RNA	899	523	1.72	0.729
TRIM34	Tripartite motif-containing protein 34	**5′ Capped RNA**	**108**	**65**	**1.67**	**<0.001**
		Poly(A)+ RNA	204	138	1.48	0.76
**Upregulated only in Poly(A)+ RNA**						
LEAP2	Liver-expressed antimicrobial peptide 2 Precursor	**5′ Capped RNA**	**139**	**98**	**1.42**	**0.003**
		Poly(A)+ RNA	626	712	2.96	0.001
LILRA5	Leukocyte immunoglobulin-like receptor subfamily A member 5 Precursor	**5′ Capped RNA**	**65**	**63**	**1.04**	**221.422**
		Poly(A)+ RNA	425	217	1.95	0.013
MAP4K2	Mitogen-activated protein kinase kinase kinase kinase 2	**5′ Capped RNA**	**121**	**94**	**1.29**	**0.037**
		Poly(A)+ RNA	482	226	2.13	0.021
PSMB4	Proteasome subunit beta type-4 Precursor	**5′ Capped RNA**	**390**	**393**	**−1.01**	**501.381**
		Poly(A)+ RNA	14358	4689	3.06	0.013
**Upregulated in both 5′ capped and Poly(A)+ RNA**						
*TRIM22	Tripartite motif-containing protein 22	**5′ Capped RNA**	**824**	**201**	**4.11**	**<0.001**
		Poly(A)+ RNA	3390	545	6.22	0.001
LAIR1	Leukocyte-associated immunoglobulin-like receptor 1 Precursor	**5′ Capped RNA**	**199**	**85**	**2.34**	**<0.001**
		Poly(A)+ RNA	337	148	2.28	0.001
**Downregulated only in 5′ capped RNA**						
IFNAR1	Interferon-alpha/beta receptor alpha chain Precursor	**5′ Capped RNA**	**312**	**646**	**−2.07**	**0.013**
		Poly(A)+ RNA	741	1220	−1.65	1.132
LILRB3	Leukocyte immunoglobulin-like receptor subfamily B member 3 Precursor	**5′ Capped RNA**	**83**	**138**	**−1.66**	**<0.001**
		Poly(A)+ RNA	922	980	−1.06	401.425

Pol II RNA transcripts were isolated from HCV cirrhotic and control liver via two methods. 5′ capped RNA was isolated using a high affinity variant eIF4E protein, poly(A)+ RNA was isolated with oligo-dT (Qiagen) and cDNA was synthesized with random primers (Methods). RNA transcript expression was measured by averaging fluorescent signal intensity on Agilent's ENCODE gene arrays for each sample (Methods). Differences in gene expression were visualized using Genoviz's Integrated Genome Browser software. Only annotated genes with ≥1.5 fold and Bonferroni corrected p values <0.05 between HCV cirrhotic and control liver are listed with details of their expression. Genes are listed by the specific pool of RNA analyzed. No genes with decreased expression in HCV cirrhotic liver were found in the poly(A)+ RNA. Genes which have been previously documented to have increased expression in HCV cirrhotic liver are marked with *. Only genes associated with the immune response are listed above. A complete list of differentially expressed genes is provided in supplemental materials.

**Figure 3:**
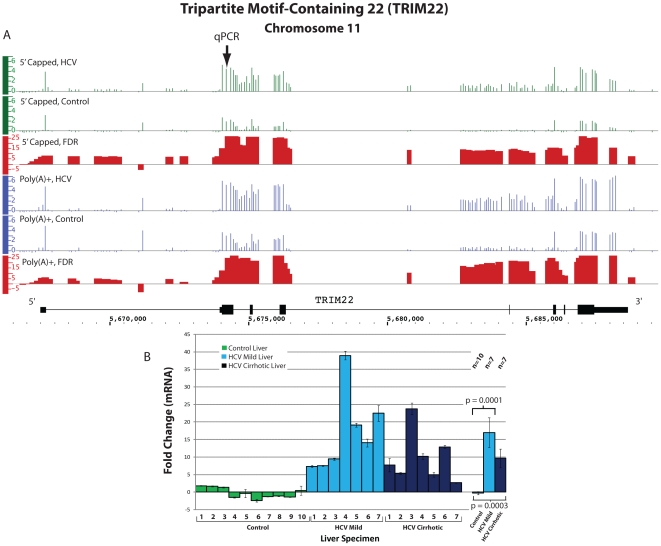
Differential expression of *TRIM22* in HCV cirrhotic as compared to control liver tissue. **Panel A.** The expression of *TRIM22* as measured by signal intensity on ENCODE tiling arrays are displayed using the IGB. The data are displayed as in Figure 2. **Panel B.** qPCR was performed as described for Figure 2 and in Methods.

The tripartite motif-containing 22 (*TRIM22*) mRNA, encoded by an interferon regulated gene previously reported to be upregulated with HIV and hepatitis B viral infections [27], [28], was also found to have increased expression in our analysis of HCV cirrhotic liver. TRIM22 was increased on ENCODE analysis in both 5′ capped (fold change 4.1, p<0.001) and poly(A)+ RNA (fold change 6.2, p = 0.001, Figure 3A); we also identified increased transcription in intronic regions within the *TRIM22* gene. qPCR analysis of *TRIM22* showed a large increased expression (fold change 9.7, p<0.001) in HCV cirrhotic liver (Figure 3B). Liver biopsies from patients with mild chronic hepatitis C and no fibrosis showed a marked increase in *TRIM22* expression (fold change 17.0, p<0.001, Figure 3B).

Connective tissue growth factor (*CTGF*) mRNA is upregulated during liver fibrosis and its protein product has recently been suggested as a non-invasive biomarker of liver fibrosis in patients infected with hepatitis C [29]. *CTGF* mRNA was upregulated in both 5′ capped (fold change 5.5, p<0.001) and poly(A)+ RNA (fold change 13, p<0.001, Figure S1A). qPCR analysis of liver tissue from multiple control and HCV cirrhotic liver specimens showed a large mean increase in *CTGF* expression (fold change 17, p = 0.004) (Figure S1B). Interestingly, qPCR analysis of liver biopsies with mild chronic hepatitis C with no fibrosis showed no increase in *CTGF* expression (fold change −5.7, p = 0.03, Figure S1B).

Several other differentially expressed genes identified in the ENCODE tiling array analysis were selected for qPCR analysis using additional patient samples. The Met proto-oncogene (*MET*) is the hepatocyte growth factor receptor and has been implicated in the development of multiple tumor types. ENCODE array analysis of 5′ capped RNA showed decreased expression of *MET* in hepatitis C cirrhotic liver (fold change −1.8, p = 0.015, Figure S2A). However, qPCR analysis of multiple patients did not demonstrate a significant difference in expression in HCV cirrhotic liver (Figure S2B). However, qPCR analysis of mild chronic hepatitis C biopsies with no fibrosis showed a significant increase in *MET* expression (fold change 3.7, p = 0.002, Figure S2B).

Cathepsin D (*CTSD*), a lysosomal aspartyl protease associated with several disease processes, was significantly upregulated in the ENCODE array analysis of 5′ capped RNA from hepatitis C cirrhotic liver (fold increase 1.9, p<0.001). However, when multiple patient samples were analyzed by qPCR no significant difference was identified (data not shown). The FUN14 domain containing 2 (*FUNDC2*) mRNA, encoding a protein suggested to interact with hepatitis C core protein, was downregulated in 5′ capped RNA from hepatitis C cirrhotic liver (fold change −2.1, p<0.001) [30]. However, when specimens of liver from additional patients with hepatitis C cirrhosis were analyzed by RT-PCR no significant difference was seen (data not shown). Nevertheless, RT-PCR of cDNA prepared from the same liver specimens (HCV cirrhotic 1 and Control 1) used in the ENCODE tiling array analysis did verify that *MET*, *CTSD* and *FUNDC2* were both significantly different in those specimens (data shown for MET only).

**Figure 4:**
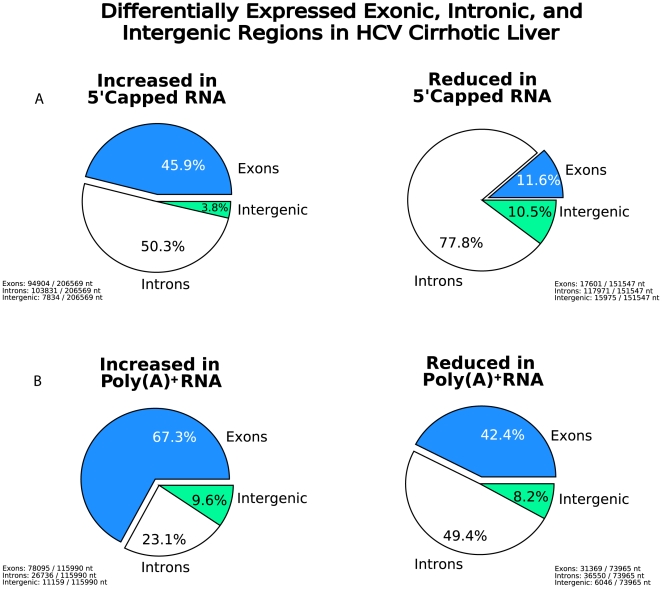
Differential gene expression observed in exonic, intronic, and intergenic regions in 5′ capped and poly(A)+ RNA. Pie charts depict the relative percentage of differentially expressed nucleotides in HCV cirrhotic liver as compared to control liver using 5′ capped RNA (**Panel** **A)** or poly(A)+ RNA analysis (**Panel** **B)**. Charts on the left show increased expression and charts on the right show reduced expression. The percentage of differentially expressed nucleotides found in intronic, exonic, and intergenic regions are shown for 5′ capped and poly(A)+ RNA samples.

Differential gene expression associated with HCV cirrhotic liver was also observed in non-protein coding genomic regions including intronic and intergenic regions. Figure 4 illustrates the differential gene expression observed in exonic, intronic, and intergenic regions in 5′ capped and poly(A)+ RNA. Fifty percent of the upregulated and 78% of the downregulated nucleotides were found in intronic regions using 5′ capped RNA, as compared to 23% of the upregulated and 49% of the reduced nucleotides when poly(A)+ RNA was analyzed. A similar percentage of differentially expressed nucleotides were observed in intergenic regions when 5′ capped (4–10%) and poly(A)+ (8–10%) RNA samples were analyzed. Four annotated genes provide examples of higher intronic expression in 5′ capped as compared to poly(A)+ RNA: hepatocyte growth factor receptor (*MET*); tissue inhibitor of metalloproteinase 3 (*TIMP3*); mitogen-activated kinase kinase kinase 1 (*MAP3K1*); MyoD family inhibitor domain containing (*MDFIC*); and (Figures S2 and S3).

**Figure 5:**
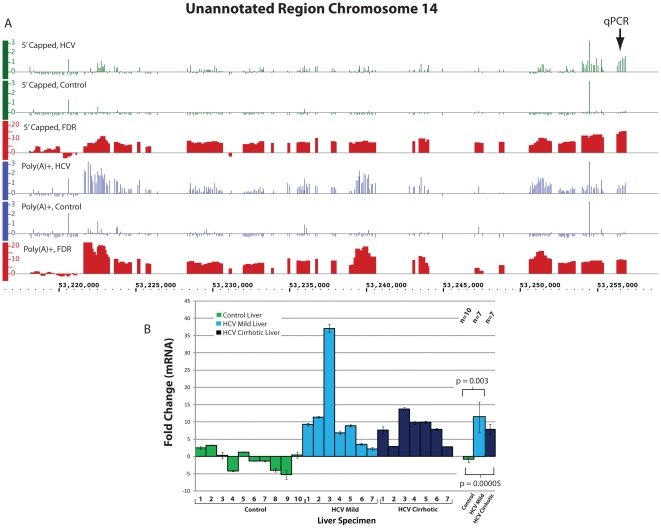
Differential expression of a Pol II RNA from an unannotated region of Chromosome 14. **Panel A.** Expression of 5′ capped and poly(A)+ RNAs as measured by signal intensity on ENCODE tiling arrays are displayed using IGB. The data is displayed as in Figure 2 except that each tick mark represents the normalized mean signal intensity of probes within a 200 nt window. **Panel** **B.** qPCR was performed as described in Methods. Triplicate samples from seven HCV cirrhotic, seven mild HCV (no fibrosis) and ten control livers were analyzed. HCV cirrhotic 1 and Control 1 refer to original samples used for the ENCODE tiling array analysis. The mean ± SEM fold change for all specimens analyzed is shown and the location of the qPCR primers is indicated by qPCR in Panel A.

**Figure 6:**
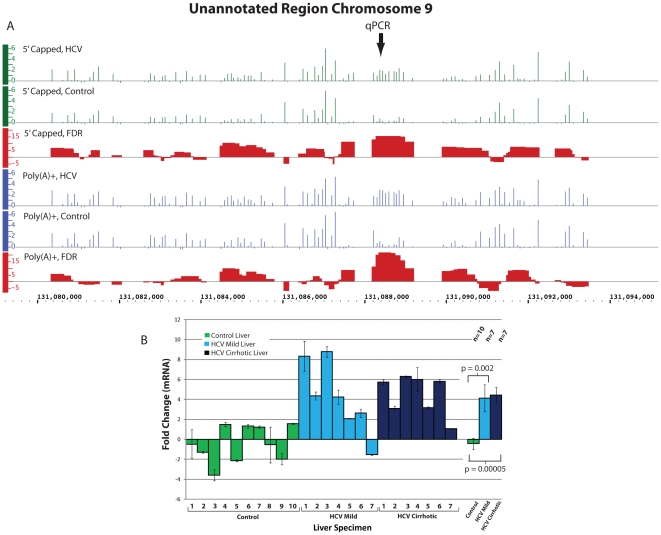
Differential expression of a Pol II RNA from an unannotated region of Chromosome 9. **Panel A.** Expression of 5′ capped and poly(A)+ RNAs as measured by signal intensity on ENCODE tiling arrays are displayed using IGB. The data is displayed as in Figure 5. **Panel** **B.** qPCR was performed as described in Methods. Triplicate samples from seven HCV cirrhotic, seven mild HCV (no fibrosis) and ten control livers were analyzed. HCV cirrhotic 1 and Control 1 refer to original samples used for the ENCODE tiling array analysis. The mean ± SEM fold change for all specimens analyzed is shown and the location of the qPCR primers is indicated by qPCR in Panel A.

Our ENCODE tiling array analysis of both 5′ capped and poly(A)+ RNA identified three differentially expressed transcriptionally active genomic regions where no annotated genes exist. These 5′ capped RNA transcript regions originated from chromosome 14 (Figure 5A, coordinates 53,254,000–53,256,899), chromosome 9 (Figure 6A, coordinates 131,088,154–131,089,262) and chromosome 21 (Figure S4A, coordinates 39,253,256–39,413,500). All three of these genomic regions demonstrated RNA transcription at least 10 kb from annotated genes. Although these regions were found to be transcribed in previous ENCODE tiling array analysis of HL60, HeLa, and Gm06990 human cell lines (Figure S5, http://genome.ucsc.edu/ENCODE/pilot.html), there was no prior evidence that they represented 5′ capped Pol II RNAs. Further investigation using the UCSC genome browser (http://genome.ucsc.edu) identified several ESTs (spliced and unspliced) originating from the transcribed region identified on chromosome 9 (Figure S6A). Not surprisingly, the 160kb region on chromosome 21 included several ESTs (BE870595, BG459638, BG460250, BI011795) and overlapped with one hypothetical protein (AJ011409, unpublished). It also included previously identified 5′ Rapid Amplification of cDNA Ends (RACE) products [31]. The 5′ capped RNA transcript(s) originating from the unannotated region of chromosome 14 did not overlap with any known genes in the UCSC database but was associated with a SNP (rs2884435) and several unspliced ESTs (Figure S6B).

qPCR analysis of the 5′ capped Pol II RNA(s) originating from the unannotated region on chromosome 14 confirmed the presence of a RNA transcript that was increased 7.8 fold in seven HCV cirrhotic as compared to ten control liver specimens (p<0.001) (Figure 5B). Moreover, qPCR analysis of mild chronic hepatitis C biopsies with no fibrosis showed a similar increase in expression of the transcript (fold change 11.3, p = 0.003, Figure 5B).

The 5′ capped RNA originating from the unannotated region on chromosome 9 was also confirmed to have a 4.5 fold increase by qPCR analysis of seven HCV cirrhotic as compared to ten control liver specimens (p<0.001) (Figure 6B). qPCR analysis of mild chronic hepatitis C with no fibrosis showed a similar increase in expression of the transcript (fold change 4.1, p = 0.002, Figure 6B).

The region on chromosome 21 did not differ significantly among multiple patients with HCV cirrhosis using qPCR.

To further define the structure of differentially expressed RNA(s) originating from chromosome 9 and 14, we used 5′ and 3′ rapid amplification of cDNA ends (RACE) and DNA sequencing over one region on chromosome 9 and three separate regions of chromosome 14 (Chr14a, Chr14b, and Chr14c) where differential expression was observed by ENCODE analysis. Using 5' RACE we identified the 5′ end of an unannotated RNA transcript found on chromosome 9 (Figure S6A). The 5′ end of this transcript was at a similar chromosome coordinate as other previously identified ESTs. Using 3′ RACE we identified the 3′ ends of independent RNA transcripts on the minus strand of Chr14a and Chr14b regions, respectively (Figure S6B). We also sequenced approximately 600–800 nucleotides including the poly(A)+ end of each transcript. The existence of at least a 1.50 kb transcript in the chromosome 14c region was confirmed by standard PCR product sequencing. To compare relative expression of RNA transcripts on the Chr14a and Chr14c regions we performed qPCR analysis. Interestingly, gene expression was similar in the HCV cirrhotic and control human liver samples but more than 10-fold higher in the Chr14a region compared to the Chr14c region in Huh7.5 cells, a human hepatoma cell line (data not shown).

## Discussion

The quantitative analysis of RNA transcripts in control and diseased tissue to define differential gene expression and aid biomarker discovery has generally used cDNA derived from total or poly(A)+ RNA hybridized to oligonucleotide microarrays. Recent studies of RNA transcription in human cells using ENCODE tiling arrays and poly(A)+ RNA have surprisingly described extensive transcription in previously unannotated genomic regions [18], [31], [32]. A consistent observation in these studies has been unexpectedly high transcription in unannotated and intronic regions of the genome. For example, a high-resolution strand-specific analysis of the entire transcriptome of *S. pombe* showed extensive transcription including intergenic and antisense transcription [19]. In addition, studies suggest that the number of Pol II transcripts with absent or short poly(A)+ ends may be markedly underestimated (ref 18, 20). Based on these findings it seems likely that standard microarray analyses are underestimating disease specific gene expression changes. In this study we tested this possibility by analyzing gene expression changes in hepatitis C (HCV) cirrhotic as compared to control liver in both 5′ capped and poly(A)+ populations of RNA using ENCODE tiling arrays representing 1% of the genome.

Differential gene expression between HCV cirrhotic and control liver, in most cases, was observed with a greater sensitivity and additional detail when 5′ capped RNA was analyzed as compared to poly(A)+ RNA (Table 1). Although many of the differentially expressed transcripts identified using poly(A)+ RNA had higher fold changes, the variability between experimental replicates was greater resulting in failed statistical testing. One possible explanation for the increased consistency in analyzing 5′ capped as compared to poly(A)+ RNA could be variability of the poly(A)+ ends. Short poly(A)+ ends have been demonstrated for a number of well-annotated genes in human liver, such as eNOS mRNA in vascular endothelial cells, and is a well-documented mechanism for regulating gene expression in developmental models [20], [33], [34]. Moreover, examples of mRNAs with a poly(A)-limiting element in their 3′ end, such as the Xenopus albumin mRNA produced by liver, have poly(A)+ ends of <20 nts yet are efficiently translated [35].

Additional differences observed in the ENCODE tiling array analysis of 5′ capped and poly(A)+ RNA populations included differences in signal intensity across specific regions of each gene transcript. In most cases the signal intensities at the 3′ end of transcripts were greater when poly(A)+ RNA was analyzed as compared to 5′ capped RNA. In some cases the signal on tiling arrays was greater in the 5′ end of annotated genes when 5′ capped RNA was analyzed as compared to poly(A)+ RNA. With some gene transcripts, such as the hepatocyte growth factor receptor (*MET*), tissue inhibitor of metalloproteinase 3 (*TIMP3*), MyoD family inhibitor domain containing (*MDFIC*) and mitogen-activated kinase kinase kinase 1 (*MAP3K1*), higher levels of transcription was observed in intronic regions when 5′ capped RNA was analyzed as compared to poly(A)+ RNA. This is consistent with the 5′ capped RNA purification that includes unspliced heterogeneous nuclear RNA (hnRNA) as well as mature spliced RNA, while the poly(A) dependent purification predominantly represents mature spliced RNA.

We identified many immune response genes that were significantly increased in HCV cirrhotic as compared to control liver, as other studies have, and found upregulated genes that were not identified previously by gene array analysis. These included *IRF1*, tripartite motif-containing 22 (*TRIM22*), and multiple leukocyte immunoglobulin-like receptors (*LILRA1, LILRA4, LILRA5, LILRB2, LILRB3* and *LILRB4*) [14], [16], [36]. IRF1 transcription was significantly increased in HCV cirrhotic as compared to control liver when 5′ capped RNA was analyzed, but not when poly(A)+ RNA was analyzed. *IRF1* is a critical transcriptional regulatory factor that modulates interferon stimulated gene (ISG) expression and has been shown to regulate HCV subgenomic replicon activity in cultured hepatoma cells [37], [38]. Interestingly, polymorphisms in the *IRF1* promoter have been reported to be associated with a better response to interferon alpha (IFN-α) therapy in patients with chronic hepatitis C [39]. Tripartite motif (TRIM) 22 was another immune response related gene that was significantly increased in HCV cirrhotic as compared to control liver in our analysis. The tripartite motif family of proteins has been associated with innate immunity to viruses by restricting viral replication [40]. *TRIM22* is dramatically upregulated by interferon signaling and decreases HIV replication (Barr et al., 2008). In addition, it has recently been shown to suppress HBV replication in culture [28]. Genome wide expression array analysis of both chimpanzee and human liver tissue have provided evidence for increased expression of TRIM22 in hepatitis C infected liver, however this is the first report to confirm its increased expression in HCV infected human liver (both cirrhotic and non-fibrotic) using real time PCR [11], [12], [41]. The qPCR analysis of biopsies showing mild hepatitis C without fibrosis provide evidence that the upregulation of *IRF1*, *TRIM22*, and *MET* are authentically due to HCV infection, and not due to major changes in liver tissue cell type.

Further evidence of alterations in immune response function includes the upregulation of multiple leukocyte immunoglobulin-like receptors. These receptors, also known as immunoglobulin-like transcripts (ILTs), are expressed on myelomonocytic cells and can influence both the innate and acquired immune response [42]. *LILRB2*, the most highly upregulated inhibitory ILT in our study, is also upregulated in HIV patients and may impair the antigen presentation of monocytes [43]. Increased expression of another inhibitory ILT, *LILRB4* or ILT3, also impairs antigen presentation and T cell recruitment as well as modulates the expression of proinflammatory cytokines [44]. Together, these transcriptional changes observed in immune response genes are consistent with viral infection. It should be noted that a number of interferon-inducible genes previously reported to be upregulated with HCV infection were not interrogated in our ENCODE tiling array analysis (representing 1% of the genome) included *STAT1*, *IRF9*, *IFI27*, *CXCL9*, *CXCL19* and *CXCL11* [41], [45], [46].

A number of gene expression studies of HCV infected liver biospecimens, done with annotated gene arrays and qPCR, have been reported. They include studies that identified potential molecular makers for HCV-associated liver disease and transcript profiles predictive of both early stage fibrosis and end stage HCV induced liver disease (cirrhosis) [14], [47]. These studies provided evidence that during late stages of HCV induced liver disease many of the changes in gene expression observed between HCV-infected liver as compared to control liver biospecimens are due to liver fibrosis and not HCV infection. Gene transcripts found to be upregulated in both early and late stage disease include many interferon-stimulated genes like *STAT1, IRF9, IFI27* and *CXCL10* while transcripts involved in growth factor signaling and tissue remodeling like *CTGF, MMP7* and *IL8* are more commonly upregulated during fibrosis [47], [48], [49]. In our tiling arrays studies, 1% of the genome was interrogated at a high degree of resolution that included approximately 400 protein coding genes. Therefore a direct comparison of gene transcripts interrogated in our dataset compared to whole annotated gene datasets is limited. Nevertheless, our gene expression results are consistent with the activation of interferon signaling pathways in both mild hepatitis C (without fibrosis) and HCV cirrhotic biospecimens as observed by the significant upregulation of both liver *IRF1* and *TRIM22* mRNA transcripts. In addition, *CTGF,* considered a biomarker of fibrosis and cirrhosis, was only upregulated in HCV cirrhotic biospecimens but not in mild hepatitis C without fibrosis similar to previous reports. With the goal of improving the selection of patients for current hepatitis C therapies, liver gene expression signatures predictive of response to therapy have been identified [13], [45], [50]. These gene signatures have included interferon stimulated transcripts including *IFI5*, *IFI6* and *IFNAR1* that are differentially expressed between responders and non-responders. Recently *IL28B* genotype has been found to be a strong predictor of response to therapy and spontaneous clearance of HCV [51], [52], [53], [54], [55]. A recent report indicates that low interferon induced gene expression in liver biopsies is a stronger predictor of treatment response than *IL28B* genotype in patients with hepatitis C [56]. The approach we describe may be useful in developing future panels of gene expression biomarkers for patients with chronic hepatitis C or other liver disorders that predict clinical outcomes such as the risk for hepatocellular carcinoma.

We also identified novel unannotated regions showing increased transcription in both HCV cirrhotic and mild hepatitis C as compared to control liver including multiple RNA transcripts on chromosome 14 and an unannotated RNA transcript on chromosome 9. It is unknown at this time whether these transcripts represent protein coding RNAs or non-coding regulatory RNAs. The use of a sequence analysis tool (BESTORF, www.softberry.com) to predict potential coding fragments in these unannotated sequences, did not identify any open reading frames greater than 40 amino acids. This finding suggests that these transcripts may represent non-coding RNAs. In addition to the presence of open reading frames (ORF), both expression level and cross-species sequence homology are important indicators of the protein coding or non-coding nature of RNA transcripts [57]. Based on the low expression level of the novel transcript found in the chromosome 14c region, it's low sequence homology to other species, and our inability to identify a poly(A)+ tail in this transcript, it may indeed represent a non-coding RNA. Although the other two RNA transcripts on chromosome 14 (14a and 14b) and one on chromosome 9 were expressed at a higher level, their low cross species homology suggests they are also non-coding RNAs. These unannotated expressed regions were evaluated for possible miRNAs using the informatics program MIREVAL [58]. Although this analysis identified multiple possible miRNA precursor hairpin structures in the chromosome 14 transcript, none of these regions encoded known miRNAs in the miRBase database [59]. Our evidence that these transcriptionally active regions represent novel Pol II transcripts is supported by transcription at these same chromosomal coordinates in published ENCODE analysis of poly(A)+ RNA from human cell lines [18]. These uncharacterized Pol II transcripts may represent genes that are part of the host antiviral defense or those required for specific steps in replication or release of mature virions. We are currently further defining these RNAs and testing their biological role in an HCV cell culture system.

We conclude that analyzing 5′ capped RNAs in eukaryotic cells and biospecimens aids the definition of the complete Pol II transcriptome and increases the sensitivity of identifying differentially expressed Pol II genes in physiological and pathophysiological states. This approach should also prove useful in identifying subsets of mRNAs that are regulated post-transcriptionally by changes in pre-mRNA splicing or 3′ polyadenylation states [33], [60], [61], [62]. Analyzing differentially selected RNAs, such as 5′ capped and poly(A)+ RNA, with next generation RNA sequencing technologies should be very useful in fully defining the Pol II transcriptome and identifying previously undefined Pol II transcripts that are differentially expressed in disease states. In addition, such novel transcripts may prove useful as biomarkers and may provide insight into the role of ncRNAs in the development and progression of specific diseases.

## Methods

### RNA isolation

Total RNA was prepared using TRIzol (Invitrogen). A single sample from one hepatitis C cirrhotic and one control liver specimen were used for the initial ENCODE tiling array analysis. A total of seven hepatitis C cirrhotic liver explants, ten control liver specimens and seven percutaneous biopsies showing mild hepatitis C with no fibrosis were used for qPCR analysis. RNA species with 5′ m^7^GpppN caps were purified using a recombinant GST fusion high-affinity variant of eIF4E (eIF4E_K119A_) which binds m^7^Gpp with a tenfold higher affinity as compared to wild-type eIF4E [23], [63]. The 5′ capped RNAs were purified as previously described using GST-eIF4E_K119A_ recombinant protein bound to glutathione-agarose beads in microfuge tubes by batch purification [20], [64]. The efficiency of this purification is 70% as compared to 30% when wild-type eIF-4E is used in such purifications (manuscript in preparation). The quantity of 5′ capped RNA was measured by NanoDrop analysis and its integrity confirmed with an Agilent 2100 Bioanalyzer. Total RNA from the same liver explants was used as a starting material to purify 5′ capped and poly(A)+ RNA. The poly(A)+ RNA was purified with oligo(dT) beads (Oligotex®, Qiagen) as previously described [20]. The quantity and integrity of poly(A)+ RNA was determined by the same measures used for 5′ capped RNA.

### Human liver pathological specimens

Liver explant pathological specimens from patients undergoing liver transplantation for chronic hepatitis C with cirrhosis (n = 7) and unused donor (control) liver tissue (n = 10) were collected with IRB approval. HCV cirrhotic liver samples were from both female and male patients as were the donor liver specimens. Percutaneous liver biopsy specimens from patients with chronic hepatitis C found to have mild inflammation and no fibrosis (mild HCV; Metavir grade 1, stage 0) (n = 7) were obtained with IRB approval. All HCV liver samples were flash frozen in liquid nitrogen generally within 5 to 10 minutes after biopsy or organ removal.

### ENCODE tiling arrays

Four experimental replicate samples of 5′ capped RNA isolated from hepatitis C cirrhotic liver and control liver were used to produce cDNA using a commercially available kit (Just cDNA Double-Stranded cDNA Synthesis Kit, Stratagene, La Jolla, CA). Random hexamers were used to prime first strand cDNA synthesis from both 5′ capped and poly(A)+ RNA samples. The cDNA product was labeled using an Agilent Genomic DNA Labeling Kit PLUS by incorporating fluorescently labeled nucleotides (Cy3-dUTP or Cy5-dUTP) using the exo-Klenow fragment. Following termination of the labeling reaction, fluorescently labeled cDNA probes were purified by isopropanol precipitation. Precipitated pellets were dried and then rehydrated in distilled water. The concentration of purified oligonucleotides was determined using a NanoDrop ND-1000 spectrophotometer. A fraction of the labeled DNA (100 ng) was assayed with an Agilent BioAnalyzer to validate the size distribution of the labeled cDNA probes.

Fluorescently labeled cDNA probes were heat denatured after being combined with cot-1 DNA, Agilent aCGH blocking agent and Agilent Hi-RPM hybridization solution. Microarray hybridizations were performed using Agilent SureHyb chambers incubated at 65°C for 40 hours with a rotational speed of 20 rpm. Following incubation, the microarray slide was washed for 5 minutes in aCGH/ChIP-on-chip Wash Buffer 1 (0.5X SSPE, 0.005% N-lauroylsarcosine; room temperature) and 5 minutes in a CGH/ChIP-on-chip Wash Buffer 2 (0.1X SSPE, 0.005% N-lauroylsarcosine; 31°C). Microarray slides (Agilent ID# 014792) were briefly dipped in a solution of acetonitrile and dried. Two microarray slides each were used for labeled cDNA generated from 5′ capped and poly(A)+ RNA from two experimental replicate samples (HCV cirrhotic and control liver). These slides were then stripped and used again with the other two experimental replicate samples.

Microarray slides were scanned in an Agilent Technologies G2505B Microarray Scanner at 5 µm resolution for the simultaneous detection of Cy-3 and Cy-5 signals. Data captured from the scanned microarray image was saved as a TIFF image file and loaded into Agilent Feature Extraction Software version 10.1.1.1. The software automatically positions a grid and finds the centroid positions of each feature on the microarray. This information was used to perform calculations that include feature intensities, background measurements and statistical analyses. Data generated by the software was recorded as tab-delimited text files which were processed using the *TiMAT2* open-source software package (http://timat2.sourceforge.net) and results were visualized and our graphics produced using the *Integrated Genome Browser* (IGB) [65].

### Bioinformatic Analysis

#### Data quality

The quality of data was assessed by calculating all pair Pearson correlation coefficients for each set of biological replicas using the unadjusted raw median intensity from the Agilent scan files (R∧2 * 100: 5′ capped hepatitis C cirrhotic: 98.6%, 85.2%, 85.4%, 89.5%, 89%, 97.7%; 5′ capped control: 97.6%, 95.7%, 90.6%, 94%, 85.3%, 90%; poly(A)+ hepatitis C cirrhotic: 93.7%, 61.3%, 82.9%, 75.1%, 91.4%, 91.1%; poly(A)+ Control: 91.2%, 91.6%, 90.1%, 91.8%, 94.5%, 94.3%). The mean R∧2*100 correlation was 89%.

#### Whole Genome Static Maps

Scaled static maps were made for comparison in *IGB* for each of the four datasets (5′ capped control, 5′ capped hepatitis C cirrhotic, poly(A)+ control, and poly(A)+ hepatitis C cirrhotic liver) using the *TiMAT2* analysis package. Agilent's ENCODE probe sequences were remapped to the NCBI 36.1 human genome build. For each sample, the four biological replica raw median probe intensities were quantile normalized and median scaled to 50 [66]. For each probe, an average was calculated for the replicas, divided by 50, and log2 transformed.

#### Whole Genome Dynamic Difference Maps

To identify regions of change between the different RNA samples (5′ capped HCV cirrhotic compared to 5′ capped control and poly(A)+ HCV cirrhotic compared to poly(A)+ control liver), a sliding window approach was taken to minimize noise using the *TiMAT2* package. The four test (HCV cirrhotic) and four control remapped raw median probe intensities were quantile-normalized and median scaled to 50. Probe level summaries were calculated by taking the log2 ratio between the mean treatment and mean control. Window level summaries were calculated by identifying 260 bp windows that contain 2 or more probes. These windows were scored by first calculating all the relative difference pairs between the treatment and the control replica probes, and second, by calculating the pseudo median of these relative difference pairs. For display purposes, the pseudo median relative difference scores were converted to log2 (ratios). To estimate window level FDRs, a null distribution of random label permutation pseudo median scored windows was created. The FDR estimation associated each real pseudo median window score was calculated by dividing the number of null distribution windows that met or exceeded the score (false positives) by the number of real windows that exceeded the score (true positives and false positives). For display purposes, the FDRs were -10Log10(FDR) transformed. This FDR estimation was used to score both enriched and reduced windows. Regions enriched (or reduced) in the HCV cirrhotic compared to control liver were created by joining overlapping and adjacent windows (max gap 200 bp) with an FDR of ≥13 (-10Log10(0.05)). The majority of such regions correspond with known annotation. To identify potentially novel transcribed regions, the window arrays were first filtered against those that intersected any known exonic sequence, using a known gene set created by combining UCSC's Known Genes and the Ensembl gene database [67], [68].

#### Gene-Centric Analysis

A near identical approach was used to identify genes differentially transcribed in the HCV cirrhotic vs. control datasets. Instead of a sliding window, normalized probe intensities falling within the exons of each gene model were compared using the pseudo median and random label statistics. Those genes with an FDR ≥13 and a log2 ratio >0.65 were considered differentially transcribed.

#### Bayesian Analysis

Differential gene expression for well-annotated genes was determined by the regularized *t*-test, which uses a Bayesian procedure [69]. Briefly, the expression level of each gene was assumed to be from a normal distribution with µ and 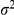. Using a conjugate prior, the mean of the posterior (MP) estimate of µ is the sample mean. The MP estimate of 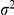 is 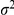 = 
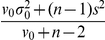
, where *n* is the sample size, *s* ^2^ is the sample variance, *ν_0_* is the degrees of freedom of the prior (a value of 10 is used in the analysis), 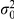 is the mean of sample variances of genes in the neighborhood of the gene under consideration. The neighborhood was the 50 genes with sample means immediately above and below the sample mean of the gene under consideration; that is, the neighborhood consists of the 101 genes centered on the gene. After the MP estimates of µ and 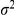 are obtained, the *t*-test of unequal variances was used to calculate a *P*-value of differential expression.

#### Real-time PCR (qPCR)

Ten control, seven hepatitis C cirrhotic liver and seven mild HCV (no fibrosis) specimens were used for further analysis of differential gene expression in hepatitis C infected liver tissue. cDNA was prepared from these pathological specimens and assayed for transcript levels of selected genes to determine if the same changes in gene expression observed in the ENCODE array analysis of one control and disease sample were observed in multiple patients with hepatitis C as compared to controls. Total RNA from control, hepatitis C cirrhotic and mild HCV liver was extracted using TRIzol followed by an RNA cleanup procedure using a RNeasy Mini kit (Qiagen, Valencia, CA). RNA was treated with DNase I (Invitrogen, Carlsbad, CA) to remove genomic DNA. First-strand cDNA was synthesized using Moloney Murine Leukemia Virus reverse transcriptase (SuperScript III; Invitrogen) with 20 ng/µl of RNA at 55°C (60 min) with random hexamers or oligo(dT) primers. Each PCR reaction was carried out in a 96-well optical plate (Roche Applied Science) in a 20 µl reaction buffer containing LightCycler 480 Probes Master Mix (100 mM Tris-HCl, 100 mM KCl, 400 µM of each dNTP (with dUTP instead of dTTP), 64 mM MgCl_2_, FastStart Taq DNA Polymerase, 0.3 µM of each primer, 0.1 µM hydrolysis probe and approximately 50 ng of cDNA (done in triplicate). Triplicate incubations without template were used as negative controls. Thermal cycling was done in a Roche LightCycler 480 System (Roche Applied Science). The qPCR thermo cycling was 95°C for 5 min, 45 cycles at 95°C for 10 sec, 59°C for 30 sec and 72°C for 1 sec. The relative quantity of each RNA transcript was calculated with the comparative Ct (cycling threshold) method using the formula 2^ΔCt^. ΔCt represents the difference between target gene expression in control samples and target gene expression in HCV samples. A reference gene (β-actin, *ACTB*) was used as the control and statistical significance was evaluated using the Student's T-test.

#### RACE Analysis

To define RNA transcript(s) structure from the differentially expressed unannotated regions on chromosomes 9 and 14, 5′ and 3′ rapid amplification of cDNA ends (RACE) were performed using cDNA made from total RNA and oligo (dT) primer or 5′ capped RNA and random primers. We designed gene specific primers on the minus and plus strand of one region on chromosome 9 and three different regions on chromosome 14 (Chr14a, Chr14b, and Chr14c) that were found to be differentially expressed in HCV cirrhotic liver by ENCODE tiling array analysis. To verify the chromosome location of each RACE PCR product, each PCR product was gel purified and cloned using a TA cloning vector. Cloned products were then sequenced with an Applied Biosystems 3130xl Genetic Analyzer.

## Supporting Information

Figure S1 Differential expression of CTGF in hepatitis C (HCV) cirrhotic as compared to control liver. Panel A. Expression of Connective tissue growth factor (CTGF) as measured by signal intensity on ENCODE tiling arrays is displayed using IGB. The data are displayed as in Figure 2. Panel B. Real-time PCR (qPCR) was performed as described in Methods. Triplicate samples from seven HCV cirrhotic, seven mild HCV (no fibrosis) and ten control livers were analyzed. HCV cirrhotic 1 and Control 1 refer to original samples used for the ENCODE tiling array analysis. The mean + SEM fold change for all specimens analyzed is shown.
(0.44 MB TIF)

Figure S2 Differential expression of MET in hepatitis C (HCV) cirrhotic as compared to control liver. Panel A. MET (mesenchymal-epithelial transition factor) is a proto-oncogene that encodes the tyrosine kinase MET and is also known as c-Met or hepatocyte growth factor receptor (HGFR). Expression of MET as measured by signal intensity on ENCODE tiling arrays is displayed using IGB. The data are displayed as in Figure 2. FDRs are depicted as negative because this gene showed less expression in hepatitis C cirrhotic as compared to control liver. Panel B. qPCR was performed as described in Methods. Triplicate samples from seven HCV cirrhotic, six mild HCV (no fibrosis), and ten control livers were analyzed. HCV cirrhotic 1 and Control 1 refer to original samples used for the ENCODE tiling array analysis. The mean + SEM fold change for all specimens analyzed is shown. Note that due to limited quantities of cDNA from mild HCV percutaneous liver biopsy specimens, duplicates were performed for four biospecimens and triplicates for two (note SEM bars for assays done in triplicate).
(0.53 MB DOC)

Figure S3 Increased intronic RNA expression from 5′ capped RNA compared to poly(A)+ RNA in HCV cirrhotic and normal human liver. Panel A, Tissue inhibitor of metalloproteinase 3 (TIMP3); Panel B, Mitogen-activated protein kinase kinase kinase 1 (MAP3K1); and Panel C, MyoD family inhibitor domain containing (MDFIC) gene transcripts. Expression of 5′ capped and poly(A)+ RNAs as measured by signal intensity on ENCODE tiling arrays are displayed using IGB.
(1.36 MB TIF)

Figure S4 Differential expression of a Pol II RNA transcript(s) originating from an unannotated region of Chromosome 21 in HCV cirrhotic as compared to control liver. Panel A. Expression of the unannotated region identified by signal intensity on ENCODE tiling arrays is displayed using IGB. The data are displayed as in Figure 5. Panel B. Liver specimens from seven HCV cirrhotic and seven control livers were analyzed by qPCR in triplicate. HCV cirrhotic 1 and Control 1 refer to original samples used for the ENCODE tiling array analysis. The mean + SEM fold change for all specimens analyzed are shown for the qPCR1 primer set. Results for the second primer set (qPCR2) also did not show a significant difference between HCV cirrhotic and control specimens (not shown).
(1.49 MB TIF)

Figure S5 Differentially expressed unannotated genomic regions in HCV cirrhotic liver compared with ENCODE data from human cell lines. The data from high density tiling array analysis of GM06690 cells (nontumorigenic B lymphocytes), HeLa cells, and HL60 (human promyelocytic leukemia, predominantly neutrophilic promyelocyte precursors) cells was loaded into IGB and aligned with the ENCODE tiling array data that we obtained in this study. The signal intensity on the ENCODE tiling arrays are displayed in IGB as in Figure 5. The aligned data provide evidence that the changes in RNA signals observed in HCV cirrhotic liver as compared to control liver in the unannotated region of chromosome 14, 9, and 21 were also observed in the ENCODE array analysis of GM06690, HL60, and HeLa cells (http://genome.ucsc.edu/ENCODE/pilot.html). The strongest signals were observed in the GM06690 cells suggesting that at least some of the signal in this region observed in HCV cirrhotic liver was due to lymphoid cells that home to and infiltrate the liver during chronic hepatitis C.
(2.11 MB TIF)

Figure S6 Structural characterization of differentially expressed unannotated Pol II transcripts on chromosome 9 and 14. Schematic drawing showing an unannotated RNA transcript on chromosome 9 and 14 identified by Agilent ENCODE tiling array analysis of 5′ capped (green bars) and polyA+ (blue bars) RNA. Panel A. Chromosome 9 The solid green block represents sequenced 5′ RACE product, with the 5′ capped end shown in black. The arrow at the top of the figure depicts location of qPCR assay. Human ESTs are depicted in solid black blocks in the format of the UCSC Genome Browser. The 5′ end of multiple ESTs on the plus strand line up with the 5′ end of our 5′ RACE product supporting the existence of a novel RNA transcript in this region. Repeating elements, also shown in black, depict highly repetitive nucleotide sequences not tiled on the ENCODE array. Affymetrix ENCODE tiling array data from a lymphoblastoid cell line (GM06990, ENCODE pilot project) is presented at the bottom of the figure (purple bars). Panel B. Chromosome 14 Three differentially expressed regions upregulated in HCV cirrhotic liver (14a, 14b, and 14c) are shown in the format of the Integrated Genome Browser (IGB). Solid green blocks show regions of sequenced 3′ RACE and PCR products, with the poly(A)+ ends shown in red. Two distinct transcripts on the minus strand of 14a and 14b regions, respectively, were confirmed by DNA sequencing. One transcript 1.5 kb in length was confirmed by DNA sequencing in the 14c region. Two black lines at the top of the figure depict location of qPCR assays. Human ESTs are depicted in solid black blocks in the format of the UCSC Genome Browser. Affymetrix ENCODE tiling array data from a lymphoblastoid cell line (GM06990, ENCODE pilot project) is presented at the bottom of the figure (purple bars).
(0.97 MB TIF)

Table S1 Upregulated genes in HCV cirrhotic liver, identified only by analyzing 5′ capped RNA. RNA transcripts were isolated from hepatitis C infected and control liver as described in Methods. cDNA was prepared using random hexamers and probes prepared as described in Methods. RNA transcript expression was measured by averaging fluorescent signal intensity on Agilent ENCODE arrays for each sample. Only annotated genes with >1.5 fold differences and Bonferoni corrected p-values <0.05 between hepatitis C infected and control liver are listed. Differentially expressed genes are categorized by function. Mean signal intensity, fold change, and p-values for each gene as determined by analyzing poly(A)+ RNA is included for comparison. Genes that have been previously documented to have increased expression in HCV infected liver are highlighted marked with*.
(0.04 MB DOCX)

Table S2 Upregulated genes in HCV cirrhotic liver identified only by analyzing poly(A)+ RNA. Annotated genes tiled on ENCODE arrays with >1.5 fold change and Bonferoni corrected p-values <0.05 are listed by function. Genes that have been previously documented to have increased expression in HCV infected liver, hepatocellular carcinoma, or cirrhosis due to other causes are marked with *.
(0.04 MB DOCX)

Table S3 Upregulated genes in HCV cirrhotic liver identified by analyzing both 5′ capped and poly(A)+ RNA. Annotated genes with a >1.5 fold change and Bonferoni corrected p-value <0.05 are listed by function. Genes that have been previously reported to be increased in HCV infected liver, hepatocellular carcinoma or cirrhosis due to other causes are marked with*.
(0.04 MB DOCX)

Table S4 Downregulated genes in HCV cirrhotic liver identified only by analyzing 5′ capped RNA. Annotated genes with a >1.5 fold change and Bonferoni corrected p-values <0.05 are listed by function. Genes that have been previously reported to be changed in hepatitis C infected liver, hepatocellular carcinoma, or cirrhosis are marked with *.
(0.04 MB DOCX)

Table S5 Downregulated genes in HCV cirrhotic liver identified only by analyzing poly(A)+ RNA. Annotated genes with a >1.5 fold change and Bonferoni corrected p-values <0.05 are listed by function.
(0.03 MB DOCX)

Table S6 Downregulated genes in HCV cirrhotic liver identified by analyzing both 5′ cap and poly(A)+ RNA. Annotated genes with a >1.5 fold change and Bonferoni corrected p-values <0.05 are listed by function.
(0.03 MB DOCX)
