# Supplementary figures and images for: ENCODE Tiling Array Analysis Identifies Differentially Expressed Annotated and Novel 5′ Capped RNAs in Hepatitis C Infected Liver
Source: PLoS One. 2011 Feb 16;6(2):e14697. doi: 10.1371/journal.pone.0014697 (PMC3040182; doi:10.1371/journal.pone.0014697)

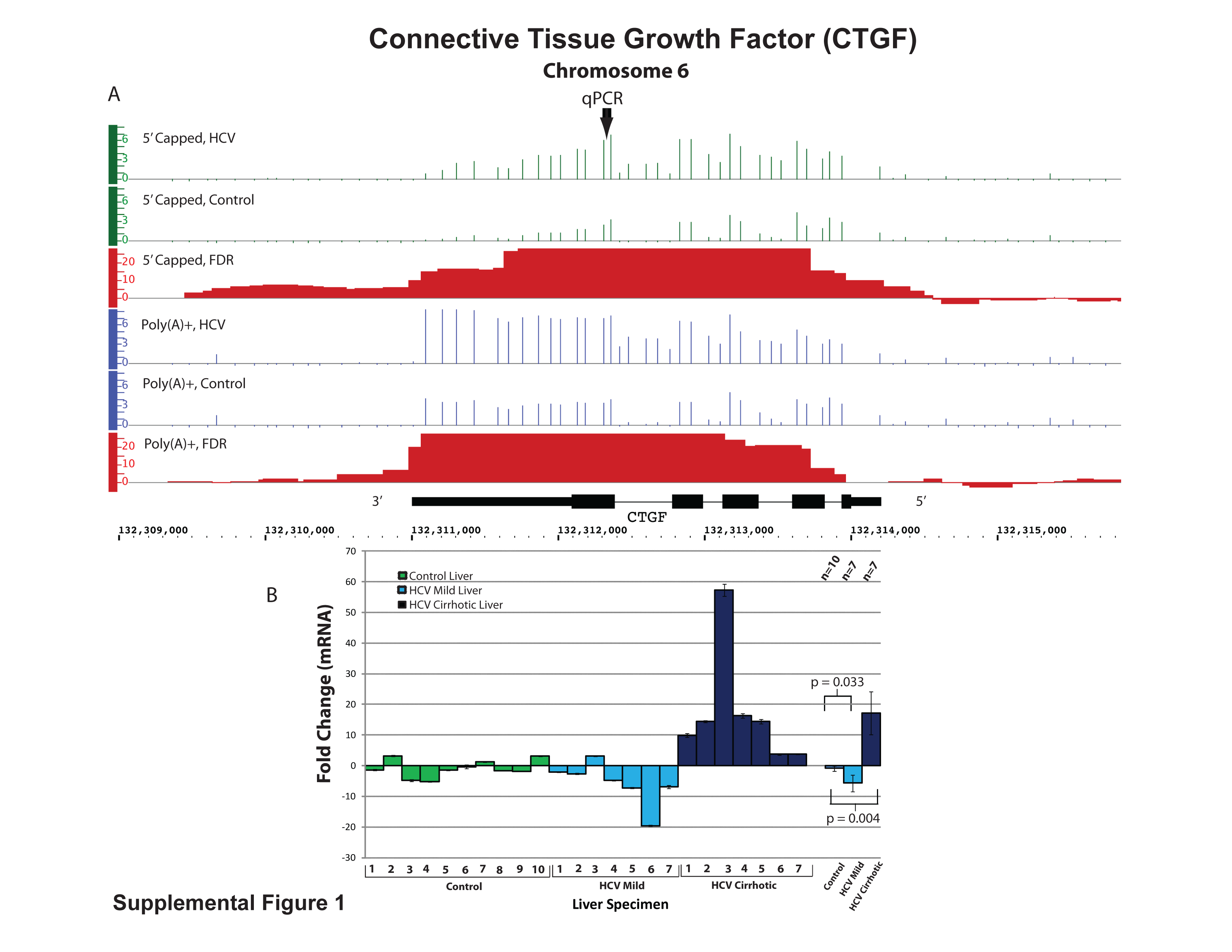

Supplement: Figure S1 — Differential expression of CTGF in hepatitis C (HCV) cirrhotic as compared to control liver. Panel A. Expression of Connective tissue growth factor (CTGF) as measured by signal intensity on ENCODE tiling arrays is displayed using IGB. The data are displayed as in Figure 2. Panel B. Real-time PCR (qPCR) was performed as described in Methods. Triplicate samples from seven HCV cirrhotic, seven mild HCV (no fibrosis) and ten control livers were analyzed. HCV cirrhotic 1 and Control 1 refer to original samples used for the ENCODE tiling array analysis. The mean + SEM fold change for all specimens analyzed is shown. (0.44 MB TIF) [file pone.0014697.s001.tif]

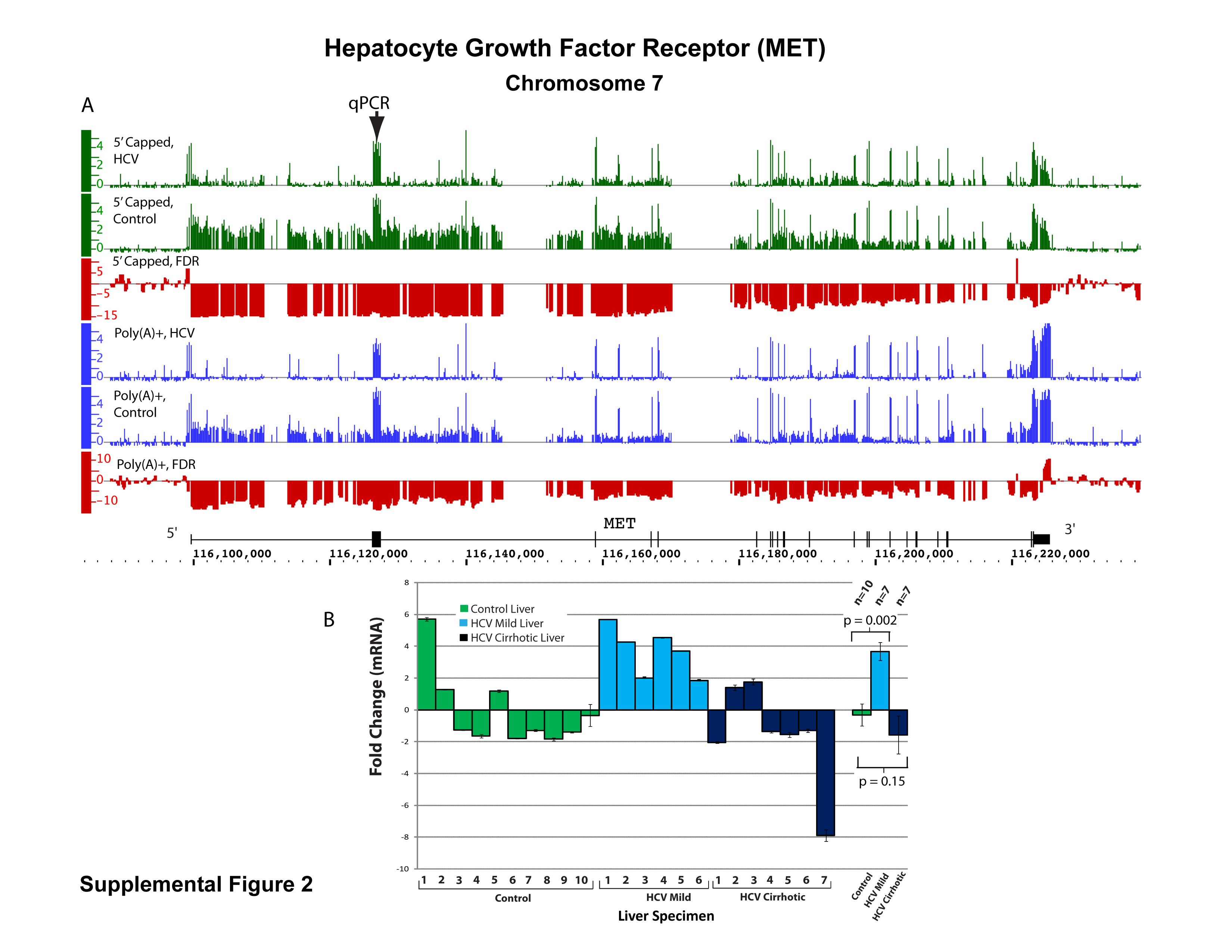

Supplement: Figure S2 — Differential expression of MET in hepatitis C (HCV) cirrhotic as compared to control liver. Panel A. MET (mesenchymal-epithelial transition factor) is a proto-oncogene that encodes the tyrosine kinase MET and is also known as c-Met or hepatocyte growth factor receptor (HGFR). Expression of MET as measured by signal intensity on ENCODE tiling arrays is displayed using IGB. The data are displayed as in Figure 2. FDRs are depicted as negative because this gene showed less expression in hepatitis C cirrhotic as compared to control liver. Panel B. qPCR was performed as described in Methods. Triplicate samples from seven HCV cirrhotic, six mild HCV (no fibrosis), and ten control livers were analyzed. HCV cirrhotic 1 and Control 1 refer to original samples used for the ENCODE tiling array analysis. The mean + SEM fold change for all specimens analyzed is shown. Note that due to limited quantities of cDNA from mild HCV percutaneous liver biopsy specimens, duplicates were performed for four biospecimens and triplicates for two (note SEM bars for assays done in triplicate). (0.53 MB DOC) [file pone.0014697.s002.tif]

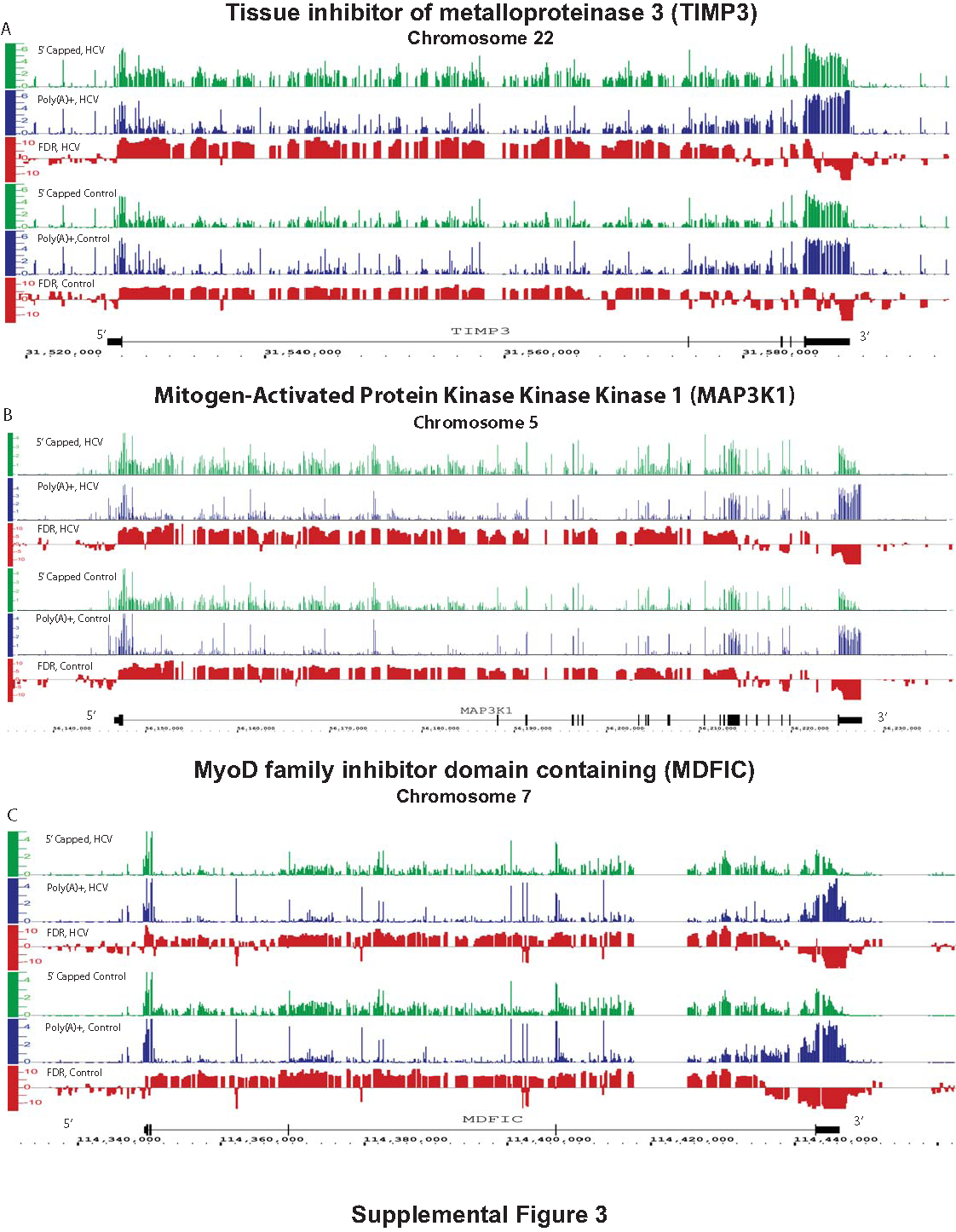

Supplement: Figure S3 — Increased intronic RNA expression from 5′ capped RNA compared to poly(A)+ RNA in HCV cirrhotic and normal human liver. Panel A, Tissue inhibitor of metalloproteinase 3 (TIMP3); Panel B, Mitogen-activated protein kinase kinase kinase 1 (MAP3K1); and Panel C, MyoD family inhibitor domain containing (MDFIC) gene transcripts. Expression of 5′ capped and poly(A)+ RNAs as measured by signal intensity on ENCODE tiling arrays are displayed using IGB. (1.36 MB TIF) [file pone.0014697.s003.tif]

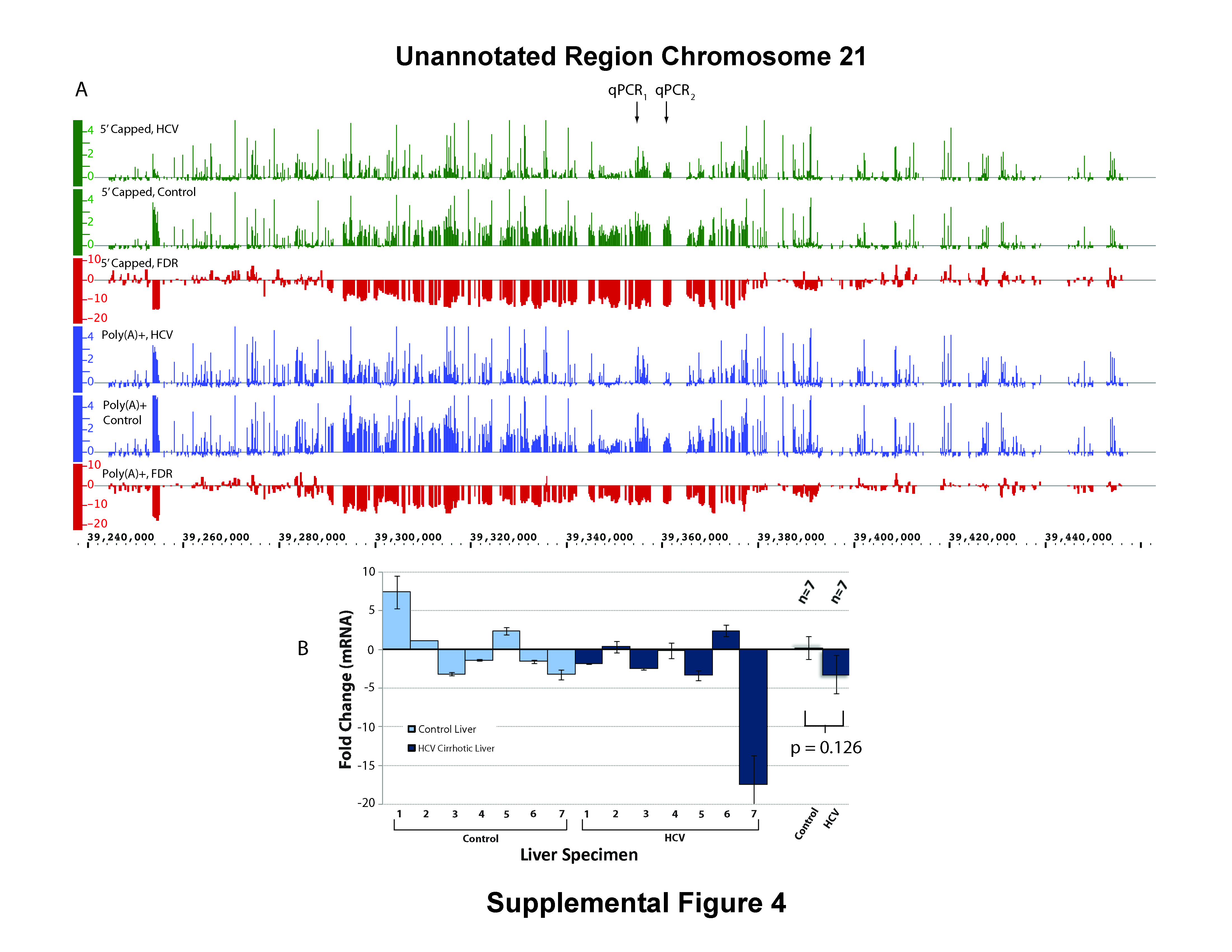

Supplement: Figure S4 — Differential expression of a Pol II RNA transcript(s) originating from an unannotated region of Chromosome 21 in HCV cirrhotic as compared to control liver. Panel A. Expression of the unannotated region identified by signal intensity on ENCODE tiling arrays is displayed using IGB. The data are displayed as in Figure 5. Panel B. Liver specimens from seven HCV cirrhotic and seven control livers were analyzed by qPCR in triplicate. HCV cirrhotic 1 and Control 1 refer to original samples used for the ENCODE tiling array analysis. The mean + SEM fold change for all specimens analyzed are shown for the qPCR1 primer set. Results for the second primer set (qPCR2) also did not show a significant difference between HCV cirrhotic and control specimens (not shown). (1.49 MB TIF) [file pone.0014697.s004.tif]

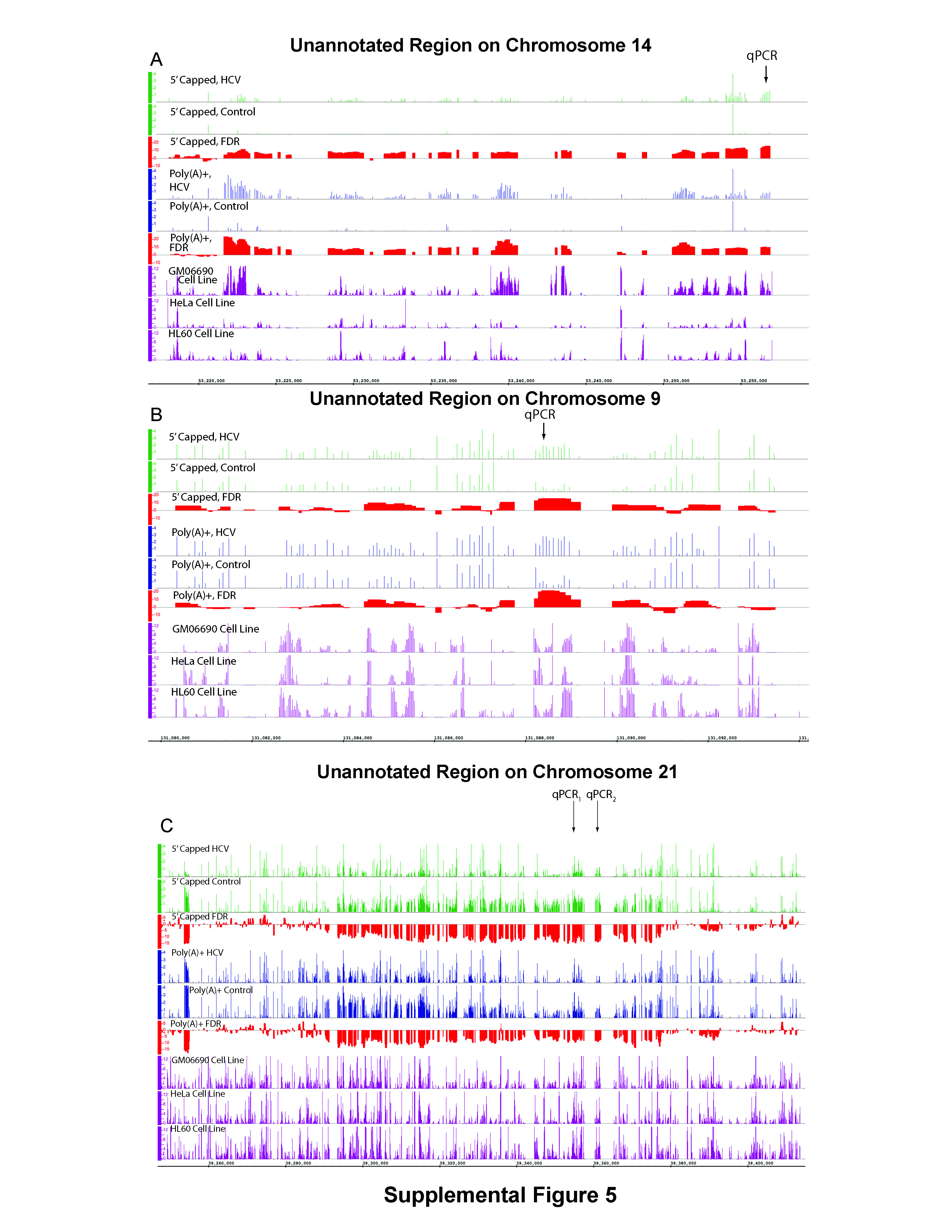

Supplement: Figure S5 — Differentially expressed unannotated genomic regions in HCV cirrhotic liver compared with ENCODE data from human cell lines. The data from high density tiling array analysis of GM06690 cells (nontumorigenic B lymphocytes), HeLa cells, and HL60 (human promyelocytic leukemia, predominantly neutrophilic promyelocyte precursors) cells was loaded into IGB and aligned with the ENCODE tiling array data that we obtained in this study. The signal intensity on the ENCODE tiling arrays are displayed in IGB as in Figure 5. The aligned data provide evidence that the changes in RNA signals observed in HCV cirrhotic liver as compared to control liver in the unannotated region of chromosome 14, 9, and 21 were also observed in the ENCODE array analysis of GM06690, HL60, and HeLa cells (http://genome.ucsc.edu/ENCODE/pilot.html). The strongest signals were observed in the GM06690 cells suggesting that at least some of the signal in this region observed in HCV cirrhotic liver was due to lymphoid cells that home to and infiltrate the liver during chronic hepatitis C. (2.11 MB TIF) [file pone.0014697.s005.tif]

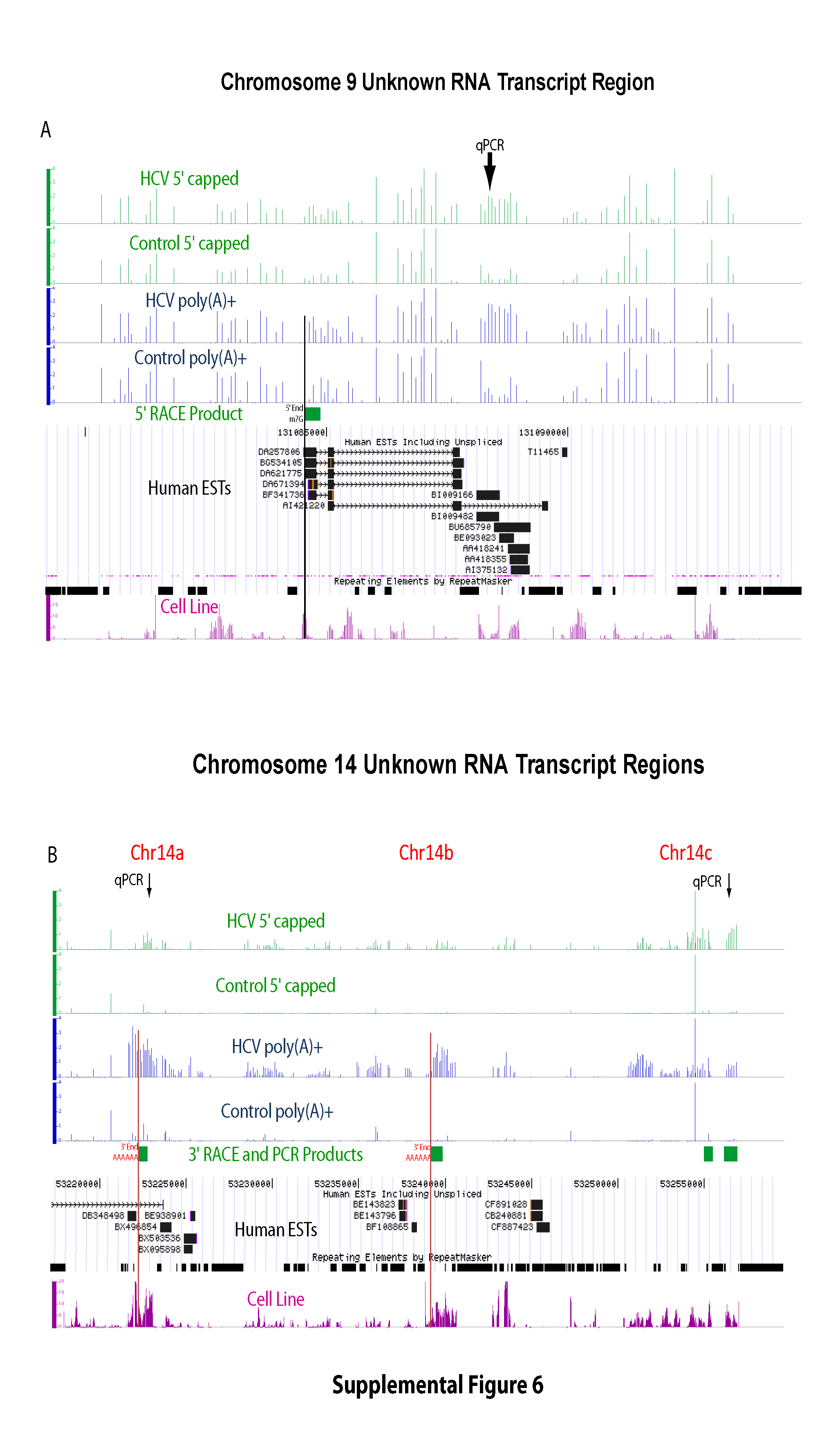

Supplement: Figure S6 — Structural characterization of differentially expressed unannotated Pol II transcripts on chromosome 9 and 14. Schematic drawing showing an unannotated RNA transcript on chromosome 9 and 14 identified by Agilent ENCODE tiling array analysis of 5′ capped (green bars) and polyA+ (blue bars) RNA. Panel A. Chromosome 9 The solid green block represents sequenced 5′ RACE product, with the 5′ capped end shown in black. The arrow at the top of the figure depicts location of qPCR assay. Human ESTs are depicted in solid black blocks in the format of the UCSC Genome Browser. The 5′ end of multiple ESTs on the plus strand line up with the 5′ end of our 5′ RACE product supporting the existence of a novel RNA transcript in this region. Repeating elements, also shown in black, depict highly repetitive nucleotide sequences not tiled on the ENCODE array. Affymetrix ENCODE tiling array data from a lymphoblastoid cell line (GM06990, ENCODE pilot project) is presented at the bottom of the figure (purple bars). Panel B. Chromosome 14 Three differentially expressed regions upregulated in HCV cirrhotic liver (14a, 14b, and 14c) are shown in the format of the Integrated Genome Browser (IGB). Solid green blocks show regions of sequenced 3′ RACE and PCR products, with the poly(A)+ ends shown in red. Two distinct transcripts on the minus strand of 14a and 14b regions, respectively, were confirmed by DNA sequencing. One transcript 1.5 kb in length was confirmed by DNA sequencing in the 14c region. Two black lines at the top of the figure depict location of qPCR assays. Human ESTs are depicted in solid black blocks in the format of the UCSC Genome Browser. Affymetrix ENCODE tiling array data from a lymphoblastoid cell line (GM06990, ENCODE pilot project) is presented at the bottom of the figure (purple bars). (0.97 MB TIF) [file pone.0014697.s006.tif]
