# Supplementary material for: ENCODE Tiling Array Analysis Identifies Differentially Expressed Annotated and Novel 5′ Capped RNAs in Hepatitis C Infected Liver
Source: PLoS One. 2011 Feb 16;6(2):e14697. doi: 10.1371/journal.pone.0014697 (PMC3040182; doi:10.1371/journal.pone.0014697)
Supplement: Table S1 — Upregulated genes in HCV cirrhotic liver, identified only by analyzing 5′ capped RNA. RNA transcripts were isolated from hepatitis C infected and control liver as described in Methods. cDNA was prepared using random hexamers and probes prepared as described in Methods. RNA transcript expression was measured by averaging fluorescent signal intensity on Agilent ENCODE arrays for each sample. Only annotated genes with >1.5 fold differences and Bonferoni corrected p-values <0.05 between hepatitis C infected and control liver are listed. Differentially expressed genes are categorized by function. Mean signal intensity, fold change, and p-values for each gene as determined by analyzing poly(A)+ RNA is included for comparison. Genes that have been previously documented to have increased expression in HCV infected liver are highlighted marked with*. (0.04 MB DOCX) [file pone.0014697.s007.docx]

**Table S1**

Poly(A)+ RNA
