# Supplementary material for: ENCODE Tiling Array Analysis Identifies Differentially Expressed Annotated and Novel 5′ Capped RNAs in Hepatitis C Infected Liver
Source: PLoS One. 2011 Feb 16;6(2):e14697. doi: 10.1371/journal.pone.0014697 (PMC3040182; doi:10.1371/journal.pone.0014697)
Supplement: Table S4 — Downregulated genes in HCV cirrhotic liver identified only by analyzing 5′ capped RNA. Annotated genes with a >1.5 fold change and Bonferoni corrected p-values <0.05 are listed by function. Genes that have been previously reported to be changed in hepatitis C infected liver, hepatocellular carcinoma, or cirrhosis are marked with *. (0.04 MB DOCX) [file pone.0014697.s010.docx]

**Table S4**

Poly(A)+ RNA
