# Supplementary material for: ENCODE Tiling Array Analysis Identifies Differentially Expressed Annotated and Novel 5′ Capped RNAs in Hepatitis C Infected Liver
Source: PLoS One. 2011 Feb 16;6(2):e14697. doi: 10.1371/journal.pone.0014697 (PMC3040182; doi:10.1371/journal.pone.0014697)
Supplement: Table S6 — Downregulated genes in HCV cirrhotic liver identified by analyzing both 5′ cap and poly(A)+ RNA. Annotated genes with a >1.5 fold change and Bonferoni corrected p-values <0.05 are listed by function. (0.03 MB DOCX) [file pone.0014697.s012.docx]

**Table S6**

Poly(A)+ RNA
